# Vulnerability Within A Nursing Clinical Practice. A Qualitative Review

**DOI:** 10.1177/23779608261428745

**Published:** 2026-03-08

**Authors:** Roger Arnold Marchen, Jette Lauritzen, Monica Evelyn Kvande, Janne Brammer Damsgaard, Maria Viftrup Schneider, Charlotte Delmar, Brith Andresen, Kjersti Nesbø, Cecilie Bræin Nilsen, Maria Iversen, Kari Lislerud Smebye, Trine Lise Jansen, Adelheid H. Hillestad

**Affiliations:** 1 155319 Lovisenberg Diaconal University College, Oslo, Norway; 2 Department of Nursing, Faculty of Health Sciences, 317905 VIA University College, Aarhus, Denmark; 3 Department of Public Health, Health Faculty, 1006 Aarhus University, Aarhus, Denmark; 4 99407 VIA Library, VIA University College, Aarhus, Denmark; 5 VID, Bergen, Oslo, Norway; 6 177041 University of South-Eastern Norway (USN), Drammen, Norway

**Keywords:** Ethics, nursing clinical practice, nurse, patient, vulnerability, qualitative review

## Abstract

**Introduction:**

Vulnerability is a fundamental human condition shaped by existential interdependence and social structures. In nursing, it is experienced by both patients and nurses, influenced by care relationships, institutional norms, and ethical responsibilities. This review explores how the phenomenon of vulnerability is reflected in the research literature on nursing clinical practice, from both patient and nurse perspectives.

**Methods:**

A qualitative literature search of eight bibliographic databases (inception to 13 May 2025) identified 29 papers, assessed using the Critical Appraisal Skills Programme Qualitative Checklist (CASP-QC). Data were analysed through qualitative content analysis inspired by Graneheim, Lundman and Lindgren.

**Results:**

Socioeconomic and sociopolitical conditions shape vulnerability by influencing care needs and perceptions of healthcare and nursing. Physical changes that compromise bodily autonomy expose patients to undignified care, as loss of control over one's body can lead to embarrassment, shame, and diminished dignity. A lack of holistic care increases patient vulnerability when professionals fail to recognise patients as unique individuals. Nurses’ vulnerability is portrayed as a significant burden, shaped by personal suffering, grief, and contextual work factors. This suffering may result in emotional distancing from patients when nurses lack the courage to engage.

**Conclusion:**

Vulnerability is multifaceted, shaped by personal, relational, and sociopolitical conditions. Patients often experience vulnerability through lack of recognition of individuality and dignity, while nurses face emotional strain, knowledge gaps, ethical tensions and limited support. Vulnerability can also be viewed as a strength, fostering ethical sensitivity, moral courage and deeper nurse–patient relationships.

## Introduction

Vulnerability is a fundamental human condition shaped by existential interdependence and social structures. Within nursing, vulnerability is experienced by both patients and nurses, influenced by care relationships, institutional norms, and ethical responsibilities. For the nursing profession, the phenomenon of vulnerability has a profound impact on the delivery of care and the treatment of patients. This review explores how the phenomenon of vulnerability is reflected in the research literature on nursing clinical practice, from both patient and nurse perspectives. It is informed by the ethical framework of ethics of proximity articulated in the works of Løgstrup and Martinsen, as well as Butler's perspectives on ethics and cohabitation.

## Background

Vulnerability is a universal phenomenon; we are all vulnerable and experience it in different ways and contexts. It can be understood in a personal context, influenced by social resources, oppression and growth, or as primarily related to health and disease (Havrilla, 2017). Mergen and Akpinar (2021) approach the phenomenon from a bioethical perspective, distinguishing between ontological grounds, which address the existential meaning of the term and circumstantial grounds, which relate to individual and social structures. Sanchini et al. (2022), in exploring vulnerability in elderly care, identified two perspectives: universal vulnerability – an ontological condition representing basic human vulnerability – and situational vulnerability, which arises from unfair social, political and economic conditions.

Inspired by Løgstrup's view of human life as one of interdependence, Martinsen (2003, 2012) argues that human beings are interconnected and dependent on one another, particularly on caring for and from others. Martinsen (2006) explains that vulnerability is a fundamental prerequisite for all life, and that our inherent vulnerability brings with it an ethical responsibility to respond to the vulnerability of others (Martinsen, 2006, 2012). Similarly, the American philosopher Judith Butler (2012) views vulnerability as universal, existential and relational. Drawing on a phenomenological perspective, Butler emphasises that the lives of others are not our own, yet are connected to ours in the sense that, from the beginning, our lives depend on a world of others, constituted in and by a social world. In contrast to Løgstrup and Martinsen, Butler's perspective in a nursing context offers insight into how our shared vulnerability affects life on both individual and social levels (Hillestad et al., 2024).

According to Sellman (2005), nursing often frames patients as vulnerable, assuming they are more susceptible to harm – either because they cannot protect themselves or because they are exposed to greater risks. Delmar (2006) stated that life phenomena constitute a fundamental condition in our search for content and meaning. Delmar (2019) further explains that some phenomena are ethical in a relational sense; some are existentially life-limiting, while others are existentially life-facilitating, such as life courage. Human vulnerability overlaps with these life phenomena, encompassing both ethical and life-limiting aspects. Engaging with patients’ life phenomena by listening to their stories requires an existential approach rather than an exclusively technological or biomedical one (Delmar, 2019).

Hillestad et al.'s (2024) review shows that nurses’ organisation of daily work and their interaction with marginalised patients can exacerbate patients’ vulnerability. This vulnerability arises not only from nurses’ norms and values but also from the structure of the institutional healthcare system. The review also highlights nurses’ own vulnerability in relation to working conditions, societal recognition and professional socialisation during nursing education.

The application of the concept of vulnerability in bioethical literature and nursing research has been questioned when used to describe the care of vulnerable individuals (Havrilla, 2017; Mergen & Akpınar, 2021; Sanchini et al., 2022). Wrigley (2015) criticises the concept as being too vague, overly broad or too narrowly defined. This review does not aim to analyse the concept itself but rather to examine the phenomenon it seeks to capture, a distinction worth noting. To the best of our knowledge, no qualitative review has synthesised the phenomenon of vulnerability in nursing clinical practice from both patient and nurse perspectives.

## Aim

This review aimed to explore how the phenomenon of vulnerability is reflected in the research literature on nursing clinical practice, from both patient and nurse perspectives.

## Methods

### Study Design

This qualitative review was developed according to updated PRISMA guidelines (Page et al., 2021). We also used the ENTREQ-guideline to ensure scientific rigour and transparency (Tong et al., 2012).

### Search Methods

In May 2025, two authors (Lauritzen and Schneider) conducted a literature search to identify studies using various research designs, such as qualitative and mixed methods studies, when qualitative data could be separated. The search strategy and terms were developed and agreed upon by all authors. The search was peer reviewed by two academic librarians and published in the Open Science Framework (OSF) Research Data Archive (Schneider et al., 2024).

### Search Strategy/Identifying Relevant Studies

The inclusion and exclusion criteria and search terms were guided by the SPIDER framework (Sample, Phenomenon of Interest, Design, Evaluation and Research type) (Cooke et al., 2012). No limits were set on year of publication (Table 1).

A comprehensive search across eight appropriate and relevant databases was conducted. CINAHL was included due to its comprehensive coverage of nursing, as the main focus of this review. MEDLINE and Embase were also selected as they are the next most relevant databases for nursing literature (Gusenbauer, 2022).

A two-step search strategy was used. First, we did a limited search of CINAHL and MEDLINE. This revealed that ‘vulnerability’ and ‘nursing’ produced a vast number of irrelevant results. Vulnerability is often used as a peripherally term in abstracts – only slightly or not at all representing the study's main focus.

A second search was therefore conducted where precision was prioritized, targeting only the title field, in order to retrieve studies with a main focus on vulnerability in nursing. We searched for two SPIDER elements – ‘Vulnerability’ AND ‘Nurs*’ (truncated) across all selected databases. The databases searched from inception to 13 May 2025 were: CINAHL (EBSCO), MEDLINE (EBSCO), AMED (EBSCO), Academic Search Premier (EBSCO), APA PsycINFO (EBSCO), Scopus, Embase and Idunn (Schneider et al., 2024).

### Selection of Studies

**Figure 1.**
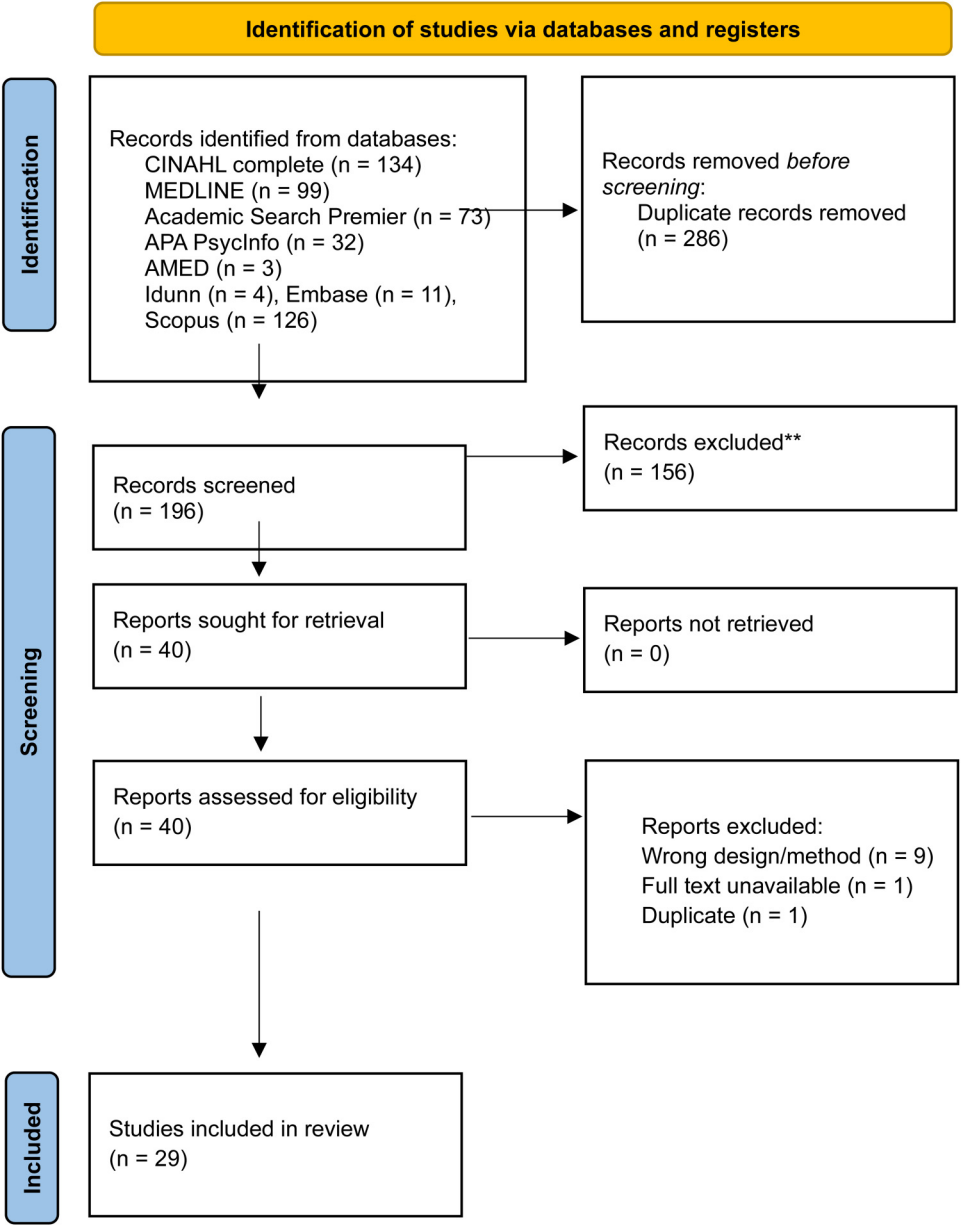
PRISMA flowchart (Page et al., 2021).

The PRISMA flowchart (Figure 1) showed that a total of 482 potentially relevant papers were identified. All identified studies were transferred to the reference management software Zotero, where 286 duplicates were removed. The titles and abstracts of the remaining 196 studies were screened in the review management software Rayyan (Qatar Computing Research Institute) by three authors (Marchen, Kvande, Hillestad) using the a priori inclusion and exclusion criteria outlined in the SPIDER framework (Cooke et al., 2012). After abstract evaluation, 156 irrelevant papers were excluded.

A full-text review of the remaining 40 papers was then conducted by the same three authors. After reading the papers, these authors discussed disagreements until a consensus was reached, and 29 papers met the inclusion criteria and were deemed eligible for the review. All included papers were independently assessed for quality using the CASP-QC (Table 2). Quality assessments were discussed among all authors until consensus was reached, after which the 29 papers were confirmed for inclusion (Table 3).

**Table 1.** Inclusion and Exclusion Criteria SPIDER Framework (Cooke et al., 2012).

	Inclusion	Exclusion
Sample (S)	Papers including nurses Papers including patients	
Phenomenon of interest (PI)	Vulnerability within a nursing clinical practice	Frailty and fragility The concept vulnerable
Design (D)	Studies with qualitative methods design. Studies with mixed methods when the qualitative data could be separated Studies available in full text	
Evaluation (E)	Perspectives and experiences of nurses and patients regarding vulnerability within a nursing clinical practice	
Research type (R)	All qualitative research of peer-reviewed studies published in scientific journals in Scandinavian or English.	Grey literature, such as books, conference proceedings and abstracts, letters, comments editorials and non-peer- reviewed papers.
Context	All clinical practices where nurses are involved	All other areas Studies exploring the experiences and perspectives of students in nursing education.
Time	No restriction on years	

**Table 2.** Critical Appraisal Skills Programme (CASP).

Authors (year of Publication)	Title	1. Was There A Clear Statement of the Aims of the Research?	2. Is A Qualitative Methodology Appropriate?	3. Was the Research Design Appropriate to Address the Aims of the Research?	4. Was the Recruitment Strategy Appropriate to the Aims of the Research?	5. Was the Data Collected in A Way That Addressed the Research Issue?	6. Has the Relationship Between Researcher and Participants Been Adequately Considered?	7. Have Ethical Issues Been Taken into Consideration?	8. Was the Data Analysis Sufficiently Rigorous?	9. Is There A Clear Statement of Findings?	10. How Valuable Is the Research?
Angel S, Vatne S, Martinsen B. (2020)	Vulnerability in Nurses: A Phenomenon That Cuts Across Professional and Private Spheres.	Yes	Yes	Yes	Yes	Yes	Yes	Yes	Yes	Yes	Valuable to some extent
Bombonatti GR, Santos DS, Marques D, Rocha FM. (2021)	Street Clinic Nursing for coping with vulnerabilities.	Yes	Yes	Yes	Yes	Yes	Can’t tell	Yes	Yes	Yes	Yes
Brandão TM, Zeviani Brêda M, Moraes Lira Nascimento YC, dos Santos de Albuquerque MC, Souza Albuquerque R. (2016)	The practices of the nurse in psychosocial care: Vulnerabilities and present potentialities.	Yes	Yes	Yes	Yes	Yes	Can't tell	Yes	Yes	Yes	Yes
Chenitz WC. (1989)	Managing Vulnerability: Nursing Treatment for Heroin Addicts.	Yes	Yes	Yes	Yes	Yes	Can't tell	Yes	Yes	Yes	Yes
Dalton ED, Pjesivac I, Eldredge S, Miller L. (2021)	From Vulnerability to Disclosure: A Normative Approach to Understanding Trust in Obstetric and Intrapartum Nurse-Patient Communication.	Yes	Yes	Yes	Yes	Yes	Can’t tell	Can’t tell	Yes	Yes	Yes
dos Santos ÉI, Gomes AMT. (2013)	Vulnerability, empowerment and knowledge: Nurses’ memories and representations concerning care	Yes	Yes	Yes	Yes	Yes	Can't tell	Yes	Yes	Yes	Yes
Ferreira SL, Cordeiro RC, Cajuhy F, Silva LS. (2013)	Vulnerabilidade de pessoas adultas com doença falciforme: Subsídios para o cuidado de enfermagem [Vulnerability in adults with sickle cell disease: Subsides for nursing care.].	Yes	Yes	Yes	Yes	Yes	Can't tell	Yes	Yes	Yes	Yes
Geuens N, Franck E, Verheyen H, De Schepper S, Roes L, Vandevijvere H, Geurden B, Van Bogaert P. (2021)	Vulnerability and stressors for burnout within a population of hospital nurses: A qualitative descriptive study.	Yes	Yes	Yes	Yes	Yes	Yes	Yes	Yes	Yes	Yes
Heaslip V, Board M. (2012)	Does nurses’ vulnerability affect their ability to care?	Yes	Yes	Yes	Yes	Yes	Can't tell	Yes	Yes	Yes	Yes
Heydarikhayat N, Ghanbarzehi N, Darban F, Kashani ZA, Rohani C. (2024)	Exploring Lived Experiences of Vulnerability in Nursing Management during the Coronavirus Disease 2019 Pandemic: A Phenomenological Study of Nurse Managers and Nurses.	Yes	Yes	Yes	Yes	Yes	Can't tell	Yes	Yes	Yes	Yes
Hudon É, Chouinard MC, Ellefsen É, Beaudin J, Hudon C. (2023)	The experience of pregnant women in contexts of vulnerability of prenatal primary nursing care: A descriptive interpretative qualitative study.	Yes	Yes	Yes	Yes	Yes	C/T	Yes	Yes	Yes	Yes
Høy B, Lillestø B, Slettebø Å, Sæteren B, Heggestad AK, Caspari S, Aasgaard T, Lohne, V, Rehnsfeldt, A, Råholm, MB, Lindwall, L, Nåden, D. (2016)	Maintaining dignity in vulnerability: A qualitative study of the residents’ perspective on dignity in nursing homes.	Yes	Yes	Yes	Yes	Yes	Can't tell	Yes	Yes	Yes	Yes
Kim MS, Kim HJ, Choi JE, Kim SJ, Chang SO. (2017)	Nursing home nurses conceptualize how to care for residents with cardiac vulnerability.	Yes	Yes	Yes	Yes	Yes	Can't tell	Yes	Yes	Yes	Yes
Liaschenko J. (1997)	Ethics and the Geography of the Nurse-Patient Relationship: Spatial Vulnerabilities and Gendered Space.	No	Can't tell	No	Can't tell	Can't tell	Can't tell	Can't tell	Can't tell	No	No
Liu YC, Chiang HH. (2017)	From vulnerability to passion in the end-of-life care: The lived experience of nurses.	Yes	Yes	Yes	Yes	Yes	Yes	Yes	Yes	Yes	Yes
Malone RE. (2000)	Dimensions of vulnerability in emergency nurses’ narratives.	Can't tell	Yes	Yes	Yes	Yes	Can't tell	Can't tell	Yes	Yes	Yes
Melissa dos Reis Pinto M, Maria Marta Nolasco C, Liliana Muller L, Laura Christina Macedo P. (2015)	The views of nurses on the vulnerability of the adolescents in a health district.	Yes	Yes	Yes	Yes	Yes	Can't tel	Yes	Yes	Yes	Yes
Morrissette P. (1986)	Avoiding the coalition trap: Recognizing the centricity and vulnerability of the psychiatric nurse in the realm of family treatment.	Yes	No	No	No	No	Can't tell	Can't tell	No	No	No
Nobis R, Sandén I. (2008)	Young men's health: A balance between self-reliance and vulnerability in the light of hegemonic masculinity.	Yes	Yes	Yes	Yes	Yes	Yes	Yes	Yes	Yes	Yes
Nugent A, Donohue G, Higgins A. (2022)	Nurses’ experiences of managing vulnerability when working with seriously ill children.	Yes	Yes	Yes	Yes	Yes	Yes	Yes	Yes	Yes	Yes
Rydeman I, Törnkvist L. (2006)	The patient's vulnerability, dependence and exposed situation in the discharge process: Experiences of district nurses, geriatric nurses and social workers.	Yes	Yes	Yes	Yes	Yes	Yes	Yes	Yes	Yes	Yes
Sarvimäki A, Stenbock-Hult B, Sundell E, Oesch-Börman C. (2017)	The vulnerability of family caregivers in relation to vulnerability as understood by nurses.	Yes	Yes	Yes	Yes	Yes	Can't tell	Yes	Yes	Yes	Yes
Silva ÍR, Gomes AMT, Valadares GV, dos Santos NLP, da Silva TP, Leite JL. (2015)	Nurses’ perceptions of the vulnerabilities to STD/AIDS in light of the process of adolescence.	Yes	Yes	Yes	Yes	Yes	Can't tell	Can't tell	Can't tell	Yes	Yes
Stenbock-Hult B, Sarvimäki A. (2011)	The meaning of vulnerability to nurses caring for older people.	Yes	Yes	Yes	Yes	Yes	Yes	Yes	Yes	Yes	Yes
Thorup CB, Rundqvist E, Roberts C, Delmar C. (2012)	Care as a matter of courage: Vulnerability, suffering and ethical formation in nursing care.	Yes	Yes	Yes	Yes	Yes	Yes	Yes	Yes	Yes	Yes
Vatne S. (2017)	Exposed to an Accumulation of Burdensome Feelings: Mental Health Nurses' Vulnerability in Everyday Encounters With Seriously Ill Inpatients.	Yes	Yes	Yes	Yes	Yes	Can't tell	Yes	Yes	Yes	Yes
Villamin P, Lopez V, Thapa DK, Cleary M, Ruishuang Z. (2025)	From Vulnerability to Stability: Migrant Nurses’ Experiences of Autonomy, Competence and Relatedness—A Qualitative Descriptive Study.	Yes	Yes	Yes	Yes	Yes	Yes	Yes	Yes	Yes	Yes
Wallerstedt B, Benzein E, Andershed B. (2011)	Sharing living and dying: A balancing act between vulnerability and a sense of security. Enrolled nurses’ experiences of working in the sitting service for dying patients at home.	Yes	Yes	Yes	Yes	Yes	Yes	Yes	Can't tell	Yes	yes
Zarth MD, Fernández PA, Baggio MA, Zilly A, Gamarra CJ, Silva RMM. (2024)	Cross-cultural nursing care for immigrant women during pregnancy and childbirth: Experiences and vulnerabilities.	Yes	Yes	Yes	Yes	Yes	Can't tell	Yes	Yes	Yes	Yes

**Table 3.** The Descriptive Characteristics of the Articles (n = 29).

Author/ Year/ Country	Aim	Methodology, Methods for Data Collection	Sample and Setting	Findings
Angel S, Vatne S, Martinsen B. (2020) Denmark	To gain insight into nurses’ experiences of vulnerability in their professional roles.	Descriptive phenomenological approach. Interviews	14 nurses Communities and hospitals	Vulnerability shows itself in feeling overwhelmed and losing bodily control
Bombonatti GR, Santos DS, Marques D, Rocha FM. (2021) Brazil	To unveil the perceptions of the Street Clinic nursing staff about coping with vulnerabilities.	Qualitative social research Participant observation with the use of a field diary and semi-structured interviews	Two nurses (and four doctors, three nursing technicians, one occupational therapist, one psychologist, one social worker, three harm reduction agents, two drivers, one administrative and one coordinator)	Living on the streets deepen health inequities through rights violating were revealed. Collaborative work, listening, and welcoming technologies stand out as mediators of a more humanized care.
Brandão TM, Zeviani Brêda M, Moraes Lira Nascimento YC, dos Santos de Albuquerque MC, Souza Albuquerque R. (2016) Brazil	To investigate the praxis of the nurse and the potentialities and vulnerabilities for this praxis in Psychosocial Care Centers (CAPS).	A descriptive study, with a qualitative approach Interviews	Seven nurses Psychosocial Care Centers of a capital of the Brazilian Northeast	Therapeutic groups, health education, individual care, home visits, medication administration and fostering were the main activities performed by nurses. Deficiencies in material resources, transportation and the structure of the service together with low professional qualifications and the weaknesses of the care network were described as vulnerabilities to work.
Chenitz WC. (1989) USA	To develop a conceptual understanding of basic psychosocial procedures used by experienced nurses working with clients during methadone maintenance.”	Grounded Theory approach. Document review, informal interviews and participant observation	209 h as a part-staff nurse, observing meetings or nurse- client interactions	The main finding is “managing vulnerability” which occurs in a therapeutic psychosocial nursing process. Through steps, it is about: “Learning to be Vulnerable”, “Living with Vulnerability”, “Beyond Vulnerability”, “Conditions for Managing Intimacy” and “Clear Clinic Policy and Effective Staff Communication.”
Dalton ED, Pjesivac I, Eldredge S, Miller L. (2021) USA	To offer conceptual clarification of the meaning of trust to providers who care for pregnant women and to answer the call to address health communication problems from a multiple goals perspective.	Quality study Open-ended interviews	22 nurses A variety of hospitals, clinics, and birth centers in three medium-sized Southeastern cities.	Trust is situated as the primary factor precluding womeńs resistance to the nurses ‘expertise, control, and authority. Trust means that the woman (1) accepts the vulnerability and risks associated with her state, (2) relinquishes control of the process to the nurse, (3) concedes that the nursés expertise is greater than her own, (4) feels as though she has a voice and is being heard, and (5) discloses all relevant information to the nurse.
dos Santos ÉI, Gomes AMT. (2013) Brazil	To analyze the interfaces among knowledge, vulnerability and empowerment present in memories and social representations concerning nursing care for carriers of HIV/Aids created by nurses.	Qualitative study with a processual approach. Sociodemographic questionnaire to characterize the subject and interviews	30 nurses Public hospital of Rio de Janeiro, a reference for the treatment of HIV/Aids and tuberculosis	Lack of professional training highlighted the vulnerability of working with patients with HIV/AIDS. Primarily, there was negative content for themes of vulnerability, such as a lack of theoretical knowledge, uncertainty and fear.
Ferreira SL, Cordeiro RC, Cajuhy F, Silva LS. (2013) Brazil	To meet some aspects related to adult living with sickle cell disease in relation to the three basic plans of vulnerability (individual, social and programmatic), pointing out some elements for nursing care.	Qualitative approach, exploratory study Interviews	12 patients A municipality that comprises the metropolitan region of Salvador	Little understanding about the disease, its implications and repercussions. Late diagnosis and lack of new therapies and small power for the transformation of attitudes and behaviours.
Geuens N, Franck E, Verheyen H, De Schepper S, Roes L, Vandevijvere H, Geurden B, Van Bogaert P. (2021) Belgium	To describe the development of nurse burnout for a population of Flemish hospital while considering the whole of vulnerability and situational stressors as indicated by the vulnerability-stress model.	Qualitative study applied a descriptive research approach Semi - structured interviews	10 nurses Hospitals and nursing specialities	Four main themes emerged: being passionate about doing well or being good, teamwork, manager, and work and personal circumstances.
Heaslip V, Board M. (2012) England	Drawing on focus group data exploring perceptions of caring for residents with dementia in a care home setting.	Pedagogical evaluation Focus groups	22 participants splitting into two focus group (FG1: six healthcare assistants, three registered nurses and one social carer. FG2: seven healthcare assistants, two registered nurses, one social carer and one trainer) Three different care homes involved in the educational programme	Feeling vulnerable, such as employees’ reaction to the disease process of dementia. Close relationship gave employees the opportunity to see the other's humanity. The disease creates grief and attention to one's own mortality.
Heydarikhayat N, Ghanbarzehi N, Darban F, Kashani ZA, Rohani C. (2024) Iran	How was the vulnerability of hospital nursing management during the spread of COVID-19?	Phenomenological approach In-depth individual semistructured interviews	14 participants (nurse managers at different levels and staff nurses)	The overarching theme of the study was “Threats to healthcare organization's management during the spread of COVID-19.” Four themes were identified as threats to nursing management within the hospital setting: “nurses attrition,” “distrust of society to the organization,” “fragility in the organization's performance,” and “intensified inequalities”.
Hudon É, Chouinard MC, Ellefsen É, Beaudin J, Hudon C. (2023) Canada	Describe factors influencing the prenatal primary nursing care experience of pregnant women in contexts of vulnerability.	Thorne's qualitative interpretative descriptive approach Semistructured interviews	21 pregnant	Financial difficulties, lack of employment, the presence of a health problem and a low level of education.
Høy B, Lillestø B, Slettebø Å, Sæteren B, Heggestad AK, Caspari S, Aasgaard T, Lohne, V, Rehnsfeldt, A, Råholm, MB, Lindwall, L, Nåden, D. (2016) Denmark	Illuminate the meaning of maintaining dignity as narrated by residents.	Phenomenological-hermeneutic approach Individual interviews.	28 patients (21 female and 7 males) Six different nursing homes, three in Norway, two in Sweden, and one in Denmark.	“Being involved as a human being, being involved as the person one is and strives to become, and being involved as an integrated member of the society.”
Kim MS, Kim HJ, Choi JE, Kim SJ, Chang SO. (2017) Korea	Identify how nursing home nurses conceptualize how to care for residents with cardiac vulnerability.	Qualitative explorative research design	30 nurses 10 nursing homes in South Korea	1 Assessing the physical, functional and cognitive conditions of residents; 2 Assessing the responses and symptoms of residents 3 using personal practice strategies based on nurses’ experience in facilities 4 Providing interventions for abnormal signs during practice 5 Following management principles of emergencies in nursing homes 6 Applying knowledge of nurses to practice.
Liaschenko J. (1997) USA	Understand the ethical concerns of home care and psychiatric nurses.	Narrative study Interviews	19 nurses (15 female and 4 males) Home care and psychiatric in San Francisco Bay Area	Two broad themes emerged: gendered space and spatial vulnerabilities. Gendered space is about the relationship with other nurses, instrumentality, and invisibility, while spatial vulnerabilities are about fragmented care, homogenizing identity, exploitation of patients for institutional gain and poverty.
Liu YC, Chiang HH. (2017) Taiwan	To explore nurses lived experiences in the provision of end-of-life (EOL) care.	Qualitative, experiential research, inductive approach to experiential research Group dialogue	13 female nurses 1800-bed military hospital in northern Taiwan	Nurses who provide EOL care experience suffering by witnessing patients’ suffering, whereas this suffering allows the nurses to authentically encounter their inner selves, consequently enables the transformation of mind-sets and further motivate them to afford and maintain passion in EOL care.
Malone RE. (2000) USA	Describe how nurses experience and cope with such vulnerability in the emergency setting and how their experiences may inform our thinking about vulnerability as a concept.	Etnographic study rooted in phenomenology Ethnographic fieldwork. Informal interviews and group interviews	40 nurses	“The emergency department as a zone of protection”, “Mythmaking as a defense against existential vulnerability”, “Distancing”, “Bearing witness to suffering” and “Existential engagement”.
Melissa dos Reis Pinto M, Maria Marta Nolasco C, Liliana Muller L, Laura Christina Macedo P. (2015) Brazil	Identify the adolescents’ vulnerabilities according to the views of nurses of a Health District. In a municipality in South of Brazil”.	Qualitative, exploratory study, based on nursing theory Praxis Intervention in Collective Health (TIPESC) Individual semi – structured interviews	16 nurses Health District	Young people do not want to go to the health centre, seek incorrect information, and avoid information about health prevention. Gender as vulnerability, with sexuality and, furthermore, pregnancy in adolescence only considered a women's problem. For men, it was more about not using a condom and engaging in drug dealing. Young people's vulnerability to sexually transmitted diseases (STDs). Lack of facilities, and violence as vulnerability.
Morrissette P. (1986) USA	Avoiding the Coalition Trap: Recognizing the Centricity and Vulnerability of the Psychiatric Nurse in the Realm of Familiy Treatment”.	Paper	Two clinical examples are presented as a picture of different coalition structures: Family-Nurse Coalition and Parents-Nurse Coalition.	Nurses are given an introductory course for the family system, and the general treatment plan is discussed with the nurses. It is encouraged to share treatment information and progress with the nurses. Nurses can observe or participate in family therapy interviews.
Nobis R, Sandén I. (2008) Sweden	To describe how young men relate to health, ill health, masculinity, and their bodies. To investigate young mens abilities to self-care.	Qualitative study Interviews	11 participants (males)	Five overall categories emerged: “body awareness”, “the creation of self-reliance”, “feelings of freedom”, “the process of self-care awareness” and “feelings of vulnerability”.
Nugent A, Donohue G, Higgins A. (2022) Republic of Ireland	Explore the experiences of children's nurses who work with seriously ill children, and to gain insight into the dynamics of working with patients and their families, as well as the nurses’ experiences of managing their own vulnerability.	Qualitative research method Semi-structured interviews	Five nurses	Three themes “being emotionally full, “navigating the rules of grief” and “prism of time”.
Rydeman I, Törnkvist L. (2006) Sweden	To achieve a deeper understanding of the experience of the discharge process among hospital nurses, district nurses, home-care nurses and social workers.	Qualitative study, a phenomenological approach Focus-group interviews	31 participants in eight focus- groups (district nurses in primary health care, district nurses/nurses in municipal-care facilities, nurses in in hospital geriatric- care units and social workers)	The patients were vulnerable, as the unique and the individual seemed not to be fully considered by the different professionals. The finding indicated that the three themes, Framework, Basic Values and Patient Resources, strongly influenced the professionals’ actions in relation to the patients in the discharge process.
Sarvimäki A, Stenbock-Hult B, Sundell E, Oesch-Börman C. (2017) Finland	To gain knowledge of the vulnerability of older family caregivers.	Qualitative approach Focus-group interviews	Older family caregivers (two female and two men aged 66–82 years)	Caregivers saw caregiving as part of being human and experienced a variety of feelings and moral agony and were harmed physically, mentally and socially. Caregivers showed courage, protected themselves and recognised that being a caregiver also was a source of maturing and developing.
Silva ÍR, Gomes AMT, Valadares GV, dos Santos NLP, da Silva TP, Leite JL. (2015) Brazil	To understand the nurses’ perception of the vulnerabilities of STD/AIDS in light of the connections of the process of adolescence.	Qualitative approach, with a Grounded Theory approach Semi - structured interviews	15 nurses University hospital in the capital of Rio de Janeiro, Brazil	Nurses perception of the vulnerabilities to STD/AIDS in light of the process of adolescence Risks and uncertainties in the process of adolescence: paths to STD/AIDS Age-adolescent complex: expanding knowledge from the perception of nurses.
Stenbock-Hult B, Sarvimäki A. (2011) Finland	To illuminate the meaning of vulnerability to providers of nursing care to older people.”	Qualitative interpretive study	16 nurses Different settings providing care for older people	The general category was being human, which is implicit in the subordinate categories: experiencing moral indignation, having feelings, having courage, being harmed, protect oneself and develop and mature.
Thorup CB, Rundqvist E, Roberts C, Delmar C. (2012) Denmark	To explore nurses’ experience of how their own vulnerability and suffering influence their ethical formation and their capacity to provide professional care when they are confronted with the patient's vulnerability and suffering.	Qualitative study Individual semi – structured interviews	23 nurses (eight from Denmark, seven from Finland and eight from Sweden) University hospitals, homecare and psychiatry hospitals	In combination with nurses’ professional qualifications and personal characteristics, ethical formation emerges over time, influenced by professional and personal experiences of suffering and of vulnerability. And either suffering and vulnerability develop into a blind spot or into an eye opener. Suffering and vulnerability also affect nurses’ courage.
Vatne S. (2017) Norway	Shed light on nurses’ vulnerability as experienced in an acute ward to gain a deeper understanding of why nurses often end up distancing themselves from being sensitive to their patients’ vulnerable behavior.	Phenomenological lifeworld perspective Field work and reflection groups	11 nurses (8 female)	Mental health nurses are exposed to feelings of mental or possible physical harm, largely than in other nursing contexts. They are not always able to protect themselves. The accumulated embodied fear of losing control, from earlier experiences, evokes in new situations and hinders nurses’ becoming sensitive to patients’ vulnerability, which in turn might escalate patients acting out. The relationship itself can, therefore, be characterized as vulnerable. Since the nurses depend on supporting staff and lack professional skills in challenging situations, they suppress the gap between their own professional caring standards and legitimated practice.
Villamin P, Lopez V, Thapa DK, Cleary M, Ruishuang Z. (2025) Australia	To understand how migrant nurses perceive their needs for autonomy, competence and relatedness are satisfied and relate how these contribute to regional workplace retention.	A qualitative descriptive study Semistructured interviews	17 migrant nurses Hospital in regional Australia.	One overarching theme, facing challenges with determination to make oneself at home, was identified, with themes: migration and relocation to a regional area, commencing and adjusting to the workplace and integrating with the community. These are further explained with subthemes: experiencing personal vulnerabilities, experiencing familial challenges and adjustment, building connections, finding one's feet, finding meaningful work through nurse empowerment, valuing relationships at work, and embracing the regional lifestyle.
Wallerstedt B, Benzein E, Andershed B. (2011) Sweden	Describe enrolled nurses’ (ENs’) experiences of working in a sitting service for dying patients at home (SSH).”	Qualitative study Focus-group interviews.	17 Enrolled nurses (EN) Municipality in the south of Sweden.	Care-giving in SSH was a balancing act between a sense of security and a feeling of vulnerability. Feeling secure and valued and that one is developing both professionally and personally, stemmed from working in partnership, whereas a feeling of vulnerability was associated with managing closeness and distance, being a mediator, having responsibility and feeling guilty, feeling hindered from doing good, facing loneliness, and affecting private lives.
Zarth MD, Fernández PA, Baggio MA, Zilly A, Gamarra CJ, Silva RMM. (2024) Brazil	To understand the experiences and vulnerabilities for cross-cultural nursing care for immigrant women during pregnancy and delivery.	Exploratory, qualitative research, in the light of the Theory of Diversity and Universality of Cultural Care Interviews	18 nurses (and eight postpartum female)	Vulnerabilities were identified in Cultural and Social Structure Dimensions expressed in access to work, low socioeconomic conditions, lack of family and social support and specific services for this population. The potentialities experienced included good care provided by health services, quality of the multidisciplinary team and appreciation of professional knowledge, however, the understanding of expectations and cultural aspects needs to be deepened.

### Analysis

The articles were analysed using qualitative content analysis inspired by Graneheim, Lundman and Lindgren (Graneheim & Lundman, 2004; Graneheim et al., 2017; Lindgren et al., 2020). First, all articles were read to gain a comprehensive understanding of the whole. The next step was to identify meaning units relevant to the study's aim. These meaning units were extracted and labelled with codes. Based on similarities and differences, the codes were grouped, re-contextualised and abstracted into categories. The categories were then grouped into four themes (Graneheim et al., 2017; Lindgren et al., 2020).

Three of the authors (Marchen, Kvande, Hillestad) conducted the initial analysis, which included reading the articles and developing codes. Data extraction was handled in Microsoft Word. They also grouped and reconstructed these codes into categories. The remaining authors reviewed the categories and themes to ensure consistency and took part in further analysis, in which sub-themes and themes were identified. Consensus was reached through dialogue within the research team, and the process continued until no further insights emerged. This approach was undertaken to strengthen the transparency and trustworthiness of the findings (Graneheim & Lundman, 2004; Graneheim et al., 2017).

## Results

The findings were organised into four themes: (a) socioeconomic and sociopolitical conditions shaping vulnerability; (b) vulnerability and the body; (c) vulnerability arising from nurse–patient interactions; and (d) nurses’ vulnerability in encounters with patients. Of the 29 articles reviewed, 26 interpret vulnerability primarily as fragility, encompassing socioeconomic disadvantage, social marginalisation, psychological insecurity, organisational pressures, and violations of dignity (Angel et al., 2020; Bombonatti et al., 2021; Brandão et al., 2016; Chenitz, 1989; Dalton et al., 2021; dos Santos & Gomes, 2013; Ferreira et al., 2013; Geuens et al., 2021; Heaslip & Board, 2012; Heydarikhayat et al., 2024; Høy et al., 2016; Hudon et al., 2023; Kim et al., 2017; Liaschenko, 1997; Liu & Chiang, 2017; Malone, 2000; Melissa dos Reis Pinto et al., 2015; Morrissette, 1986; Nobis & Sandén, 2008; Nugent et al., 2022; Rydeman & Törnkvist, 2006; Silva et al., 2015; Vatne, 2017; Villamin et al., 2025; Wallerstedt et al., 2011; Zarth et al., 2024). The remaining three articles interpret vulnerability as a strength (Sarvimäki et al., 2017; Stenbock-Hult & Sarvimäki, 2011; Thorup et al., 2012). These studies emphasise acknowledging nurses’ own vulnerability, transforming challenging situations into inner strength, and viewing vulnerability as a foundation for ethical formation, requiring courage to act, stay and speak out.

Overall, most studies interpret vulnerability as fragility linked to disadvantage and marginalisation, while a minority regard it as a resource for ethical growth and resilience.

### Socioeconomic and Sociopolitical Conditions Shaping Vulnerability

Socioeconomic and sociopolitical conditions shape vulnerability by influencing care needs and perceptions of healthcare and nursing. Disadvantages often intersect among people experiencing homelessness, low educational attainment, poverty, or financial hardship (Bombonatti et al., 2021; Brandão et al., 2016; Heydarikhayat et al., 2024; Høy et al., 2016; Hudon et al., 2023; Liaschenko, 1997; Melissa dos Reis Pinto et al., 2015; Rydeman & Törnkvist, 2006; Silva et al., 2015; Villamin et al., 2025; Zarth et al., 2024). Pregnant women in vulnerable circumstances frequently lack decision-making power, social support, and access to care, while young adults with limited education face heightened risks of STDs, HIV and AIDS. These conditions delay or prevent access to healthcare, increasing unintended pregnancies, STDs, exposure to drug trafficking, and reducing life expectancy (Hudon et al., 2023; Melissa dos Reis Pinto et al., 2015; Silva et al., 2015; Zarth et al., 2024). Homeless individuals often confront substance abuse, trafficking, and violence, perpetuating cycles of marginalisation and inadequate care for themselves and their children (Bombonatti et al., 2021). Older adults encounter particular vulnerabilities when entering nursing homes or other care facilities, where dignity, autonomy, and social engagement may diminish. Hospital discharge is another critical juncture, where systemic weaknesses, limited ethical consideration, and insufficient patient involvement can lead to increased dependence and neglect of individuality (Høy et al., 2016; Rydeman & Törnkvist, 2006).

Systemic shortcomings in mental healthcare, such as under-resourced services, inadequate training, poverty, violence, and social exclusion, further exacerbate vulnerability; fragmented care, excessive bureaucracy, and commodification of services are of particular concern to frontline nurses (Brandão et al., 2016; Liaschenko, 1997). During the COVID-19 pandemic, nursing leadership faced added challenges including staff depletion, societal distrust in healthcare organisations, fragile operational performance, and widening inequalities (Heydarikhayat et al., 2024). Migrant nurses in Australia reported vulnerability linked to financial strain and limited social networks following migration, contributing to loneliness despite efforts to adapt (Villamin et al., 2025). Overall, socioeconomic and systemic conditions create layered vulnerabilities that intersect with institutional shortcomings, reinforcing cycles of disadvantage and limiting access to dignified care.

### Vulnerability and the Body

Physical changes that compromise bodily autonomy can expose patients to undignified care, as loss of control over one's body may lead to embarrassment, shame, and diminished dignity. Dependence on others intensifies these risks, as seen among men with disabilities whose reliance on carers can threaten self-esteem and engender helplessness (Høy et al., 2016; Nobis & Sandén, 2008; Rydeman & Törnkvist, 2006).

In reproductive care, vulnerability is shaped by the dual-patient configuration of mother and foetus, uncertainty around birth, and anxiety. Where trusting relationships with nurses are present, women are more able to acknowledge vulnerability, accept risks, and disclose issues such as substance use, uncertain paternity, or abuse (Dalton et al., 2021). More broadly, adolescent sexual health risks are influenced by gender norms that shape sexual behaviours and produce disparities in exposure to STD and AIDS, highlighting how socio-cultural expectations interact with bodily vulnerability and the need for gender-responsive, trust-based care (Silva et al., 2015). Bodily vulnerability emerges when autonomy is compromised, with trust and relational care acting as key mediators in preserving dignity and reducing harm.

### Vulnerability Arising from Nurse–Patient Interactions

A lack of holistic care increases patient vulnerability when professionals fail to recognise patients as unique individuals (Heaslip & Board, 2012; Høy et al., 2016; Rydeman & Törnkvist, 2006). Nursing home residents often felt overlooked when nurses prioritised tasks over personal engagement. From the resident's perspective, dignity entails being recognised and supported as an active member of society (Høy et al., 2016). Transitions from hospital to other care settings often felt isolated and dependent, shaped by overly medicalised care (Rydeman & Törnkvist, 2006).Vulnerability is further compounded by knowledge gaps as limited professional understanding can lead to diagnostic delays and treatment challenges, illustrated by the experiences of patients with sickle cell disease and their families (Ferreira et al., 2013).

Nurses in dementia care may find disease progression distressing, leading them to focus on clinical rather than emotional care to avoid attachment (Heaslip & Board, 2012). Similarly, dos Santos and Gomes (2013) found that caring for patients with HIV/AIDS could provoke insecurity, mechanised routines, reduced self-esteem, and lower quality of care.

Vulnerability in nurse–patient interactions often stems from insufficient holistic care and knowledge gaps, with emotional distancing and task prioritisation undermining dignity and trust.

### Nurses’ Vulnerability in Encounters with Patients

Several studies portray nurses’ vulnerability as a significant burden, shaped by personal suffering, grief, and contextual work factors. Such suffering may create emotional distance from patients when the courage to engage is lacking (Thorup et al., 2012); loss and grief are associated with vicarious trauma and persistent anxiety, even in the absence of direct personal involvement (Nugent et al., 2022), while unresolved grief can erode self-esteem and compromise mental health, thereby increasing vulnerability (Liu & Chiang, 2017).

Vulnerability is associated with burnout (Geuens et al., 2021) and is heightened under the pressures of caring for older adults (Stenbock-Hult & Sarvimäki, 2011). Knowledge gaps and professional uncertainty further contribute (Angel et al., 2020; dos Santos & Gomes, 2013), though vulnerability can be mitigated through interaction with patients, relatives, colleagues, and managers (Angel et al., 2020), and by specialised knowledge in domains such as HIV/AIDS (dos Santos & Gomes, 2013). Working alone or with terminally ill patients heightens vulnerability as nurses confront their own mortality (Heaslip & Board, 2012; Wallerstedt et al., 2011), and limited bereavement support and insufficient collegial backing can hinder the processing of loss, intensifying stress (Geuens et al., 2021; Nugent et al., 2022). Ethically, the nurse's body serves as an “ethical thermometer” (Angel et al., 2020); psychiatric nurses mediate family distress while simultaneously supporting therapists (Morrissette, 1986); and vulnerability is linked to ethical formation, with courage emerging as a critical resource for helping patients face suffering and sustaining a commitment to care (Thorup et al., 2012). This courage arises from a deepened awareness of vulnerability in others (Malone, 2000). Overall, nurses’ vulnerability reflects emotional strain, ethical challenges, and knowledge gaps, while courage and collegial support remain essential for maintaining high-quality care.

## Discussion

This review explored how the phenomenon of vulnerability is reflected in the research literature on nursing clinical practice, from both patient and nurse perspectives. A dual perspective emerges, underscoring the complexity of the phenomenon. Most studies portray vulnerability as fragility; fewer show it as strength, emphasising courage.

Vulnerability as fragility includes socioeconomic disadvantage, marginalisation, psychological insecurity, and individual vulnerability overlooked in organisational contexts (Bombonatti et al., 2021; Geuens et al., 2021; Hudon et al., 2023; Morrissette, 1986). This aligns with Butler's (Butler & Berbec, 2017) concept of precarity, which describes conditions where certain populations suffer disproportionately from failing social and economic support networks.

Vulnerability as strength highlights courage and the recognition of one's vulnerability. It involves transforming challenges into inner strength and viewing vulnerability as a basis for ethical formation, with courage to act, stay and speak out (Sarvimäki et al., 2017; Stenbock-Hult & Sarvimäki, 2011; Thorup et al., 2012). Delmar (2024) discusses professional courage as essential for nurses, given the complexity and unpredictability of clinical practice. Courage is not the absence of fear but the ability to act wisely and rightly despite it. It means being present with patient suffering, challenging personal biases, tolerating rejection, trusting decisions, and prioritising professional judgement. Delmar (2024) further describes courage as an active urge to act and engage in life, fostering meaning and promoting life.

Our study found that vulnerability manifests across social groups, including those who are homeless, undereducated, or impoverished, often facing intersecting challenges (Bombonatti et al., 2021; Hudon et al., 2023; Melissa dos Reis Pinto et al., 2015; Silva et al., 2015; Zarth et al., 2024). Social, economic, and institutional factors intertwine, shaping care needs, perceptions of healthcare, and attitudes toward nurses (Bombonatti et al., 2021; Brandão et al., 2016; Heydarikhayat et al., 2024; Høy et al., 2016; Hudon et al., 2023; Liaschenko, 1997; Melissa dos Reis Pinto et al., 2015; Rydeman & Törnkvist, 2006; Silva et al., 2015; Villamin et al., 2025; Zarth et al., 2024). Sanchini et al. (2022) identified both universal and situational vulnerability in elderly care and proposed three responses: understanding vulnerability, providing care, and intervening through socio-political measures. This is supported by Havrilla (2017) who found that vulnerability involves both individual and societal factors, influenced by social resources, oppression and health. Hillestad et al. (2024) found that healthcare structures and nurses’ work organisation can exacerbate patient vulnerability. This observation is consistent with that of Butler (2004) who explained that systemic inequalities shape nursing care, calling for practices that challenge norms and prioritise dignity.

Older adults face vulnerabilities when moving to care facilities, where dignity, autonomy, and social participation are often compromised. Discharge from hospital can heighten vulnerability when systemic weaknesses and limited patient involvement foster dependence and erode individuality (Høy et al., 2016; Rydeman & Törnkvist, 2006). Lévinas (1969) frames face-to-face encounters as ethically charged, wherein exposure to the other discloses vulnerability and imposes a responsibility to respond. This aligns with our findings and resonates with Martinsen's emphasis on the challenge of sustaining cultures that acknowledge and ethically safeguard existential vulnerability (Martinsen, 2006).

Our study found that systemic shortcomings in mental health care, and excessive bureaucracy, increase vulnerability and compromise care quality. The COVID-19 pandemic further exposed weaknesses through nurse depletion, societal distrust, organisational fragility, and widening inequalities (Brandão et al., 2016; Heydarikhayat et al., 2024; Liaschenko, 1997). Hillestad et al. (2024), highlight nurses’ vulnerability in relation to working conditions, and professional socialisation during their nursing education. Similarly, Stokes-Parish et al. (2023) found that critical care nurses rejected labels such as ‘heroes’ and ‘angels’ calling for improved role representation, recognition, and safe working conditions.

Vulnerability to undignified care arises from physical changes that compromise bodily autonomy, leading to embarrassment, shame, and a diminished sense of dignity. Dependence on others heightens the risk of undignified care (Høy et al., 2016; Nobis & Sandén, 2008; Rydeman & Törnkvist, 2006). Martinsen (2006) refers to the biblical story of the Good Samaritan as a fundamental narrative in care ethics, illustrating lifès vulnerability and our dependence on one another. According to Løgstrup (1997), one never interacts with another person without some degree of control; we constitute one another's world and destiny. Martinsen emphasises that being a caring nurse is a way of being, characterised by sensitivity to human vulnerability and a commitment to alleviating suffering while safeguarding dignity and worth. This aligns with our findings and those of Høy et al. (2016), who observed that nursing home residents often felt overlooked when nurses prioritised routine tasks over personal interaction. From the residents` perspective, dignity involves being acknowledged and supported as active members of society. Delmar (2019) adds that the asymmetrical nurse–patient relationship can either expand or constrain life-conductive possibilities. To expand these possibilities, nurses must recognise their role in inherent power relations. When nurses reject their own vulnerability and keep a protective distance, care becomes paternalistic, fostering passivity and undermining person-centred practice, potentially limiting or destroying the patient's life-conductive possibilities.

Our study found that a lack of holistic perspective and failure to acknowledge patients as unique individuals contribute to vulnerability (Heaslip & Board, 2012; Høy et al., 2016; Rydeman & Törnkvist, 2006). Patients felt overlooked and devalued when nurses prioritised medical tasks over holistic needs, often leaving them isolated, exposed, and dependent (Høy et al., 2016; Rydeman & Törnkvist, 2006). These findings resonate with Martinsen's (2006) rephrasing of Løgstrup's assertion that one always holds something of another person's life in one's gaze, and through that gaze, also in one's power. An objective attitude from healthcare professionals may deepen patientś sense of isolation. This aligns with Eriksson's (2002, 2006) theory of the suffering human being, which emphasises that nurses can either promote healing and comfort or, conversely, intensify suffering and vulnerability. Caring suffering arises when nurses fail to meet patients’ needs for respect, dignity and compassionate care.

An important aspect of our findings concerns dignity in care. Maintaining dignity involves treating patients as human beings, recognising who they are, and supporting their efforts to belong and participate as integrated members of society (Høy et al., 2016; Rydeman & Törnkvist, 2006). This reflects Martinsen's (2006) assertion that, in encountering a wounded person, one should look with the ‘eye of the heart’, a participatory, attentive gaze that allows the other to emerge as significant. The ethical demand arises when one remains open and receptive, striving to understand what is at stake. Through perception and understanding, this becomes a fundamental experience of protecting and caring for life.

Lévinas (1969) discusses the ethics of human encounters, emphasising vulnerability as central to face-to-face relationships. Such encounters expose us to otherś needs and demands, revealing our inherent vulnerability and ethical responsibility to respond. Inspired by Lévinas, Butler (2004, 2005, 2012) emphasises that face-to-face encounters are foundational to our interdependence within collective and political social structures. Encountering the face of the other and grasping its meaning serves as a powerful reminder of both the otheŕs vulnerability and humanity, and of the vulnerability of life itself. For vulnerability to become a productive force, Butler argues, it must be articulated, recognised and acknowledged. Our findings suggest that nurseś vulnerability is not merely an individual trait but a relational and ethical condition shaping encounters. When the courage to engage with suffering is lacking, emotional distance may arise, leaving patients at risk of feeling unseen, a vulnerability further intensified by grief, burnout, and professional uncertainty, and limited collegial support (Angel et al., 2020; dos Santos & Gomes, 2013; Geuens et al., 2021; Liu & Chiang, 2017; Nugent et al., 2022; Stenbock-Hult & Sarvimäki, 2011; Thorup et al., 2012). Yet vulnerability can also be ethically formative. When nurses recognise vulnerability in others, courage emerges as a vital resource for continuing care and responding meaningfully to patients’ appeals (Malone, 2000; Thorup et al., 2012). This aligns with Martinsen’s view that, in clinical contexts, nurses who are sensitive and attentive become receptive, touched and moved to respond to patients’ appeals and needs. Sensitivity involves presence and engagement, allowing nurses to listen, attend to, and care for patients. Conversely, when nurses are not attentively and vulnerably present, often due to the fast pace of the healthcare system, patients may be left exposed and may withdraw from receiving support (Martinsen, 2012).

### Implications for Nursing Education and Practice

The results show that, in nursing education, curricula should integrate vulnerability as a core concept, not only as a patient characteristic but as an ethical and relational dimension of care. Vulnerability may serve as a source of strength and ethical formation, preparing nurses for reflective practice. Nursing education should also address systemic conditions, such as the social determinants of health and institutional structures that shape vulnerability, thereby equipping nurses to advocate for equity and dignity in care.

In clinical practice, nurses should adopt approaches that view vulnerability as a dynamic and relational phenomenon, fostering dignity and resilience while addressing systemic inequalities that perpetuate disadvantage. This calls for attentiveness, presence, and professional courage to uphold person-centred care under constraint, supported by work environments that allow time for relational care and prevent distancing attitudes and paternalistic practices.

The results also show that, at the policy level, policies influence organisational cultures in ways that shape values, norms, and the possibility of holistic care and ethical responsiveness, recognizing that vulnerability is inherent to human life and central to the nurse-patient relationship.

### Strengths and Limitations

A key strength of this qualitative review is its systematic and critical approach adopted throughout the review process, including the use of the PRISMA framework, the ENTREQ-guideline, and the SPIDER tool to guide inclusion criteria tailored for qualitative research.

Qualitative content analysis enabled an in-depth synthesis of complex and nuanced perspectives on vulnerability in clinical nursing practice. The inclusion of diverse clinical nursing contexts and populations enriched the understanding of how vulnerability manifests across different situations, individuals, and illness experiences. However, the review also encountered certain limitations. The number of well-indexed qualitative studies explicitly addressing vulnerability in clinical nursing practice was limited. This may reflect a broader underrepresentation of phenomenological and ethically grounded perspectives in current nursing research databases. The relatively small number of included studies may be partially a result of the restrictive search strategy rather than a true lack of existing research. The findings indicate that vulnerability as a phenomenon is relevant within clinical nursing practice, both in terms of patient experiences and the emotional and professional dimensions of nursing.

Future research should explore vulnerability from relational, ethical, and organisational perspectives to support and strengthen clinical practice and political relevance.

## Conclusion

Vulnerability is a multifaceted phenomenon that affects both patients and nurses, shaped by personal, relational and sociopolitical conditions. It is often perceived as fragility, emerging in contexts of socioeconomic hardship, illness, dependency and organisational neglect. For patients, vulnerability may arise from a lack of recognition of individuality and dignity; for nurses, it can stem from emotional distress, knowledge gaps, ethical tensions and inadequate support.

Vulnerability can also be understood as a strength, fostering ethical sensitivity, moral courage and deeper nurse–patient relationships. Holistic, person-centred and ethically conscious care is essential – recognising patients as unique human beings and viewing nurses’ vulnerability not as weakness but as a source of ethical reflection and relational depth. In nursing, vulnerability should be regarded as a dynamic, relational phenomenon that demands attentiveness, courage, and ethical responsiveness to human suffering and interdependence.
